# Paroxysmal Nocturnal Hemoglobinuria: A Case Report in a Pandemic Environment

**DOI:** 10.3390/reports6030042

**Published:** 2023-09-08

**Authors:** Vanda Peixoto, Ana Carneiro, Fernanda Trigo, Mónica Vieira, Cristina Prudêncio

**Affiliations:** 1 Chemical and Biomolecular Sciences, School of Health, Polytechnic Institute of Porto, 4200-072 Porto, Portugal; mav@ess.ipp.pt (M.V.); cprudencio@ess.ipp.pt (C.P.); 2 Center for Translational Health and Medical Biotechnology Research (TBIO), Polytechnic Institute of Porto, 4200-465 Porto, Portugal; 3 Department of Hematology, University Hospital Center of São João, 4200-319 Porto, Portugal; ana.carneiro@chsj.min-saude.pt (A.C.); ftrigo@chsj.min-saude.pt (F.T.); 4 Institute for Research and Innovation in Health (i3S), University of Porto, 4200-135 Porto, Portugal

**Keywords:** paroxysmal nocturnal hemoglobinuria, vaccines, thrombosis, eculizumab, COVID-19

## Abstract

Paroxysmal nocturnal hemoglobinuria (PNH) is a clonal, rare, complement-mediated hemolytic anemia. PNH can be associated with marrow failure and thrombophilia. We present a clinical report of splenic vein thrombosis in a patient with classic PNH. A 41-year-old male with classic PNH, naïve to complement inhibitor therapy, developed splenic vein thrombosis as a major adverse effect after vaccination protocol to prevent meningococcal disease. We also report anticoagulant and eculizumab treatment outcomes. In PNH patients, vaccination should be monitored to prevent major outcome events, like vaccine-induced thrombosis. Eculizumab proves effective for treating intravascular hemolysis and preventing more thrombotic events. The potential protective role of eculizumab on controlling complement activity and consequent inflammation may help the patient to not experience breakthrough hemolysis when infected with SARS-CoV-2. Extravascular hemolysis remains present, but new molecules are being studied to inhibit proximal complement and there is a good health prospective for PNH patients.

## 1. Introduction

Paroxysmal nocturnal hemoglobinuria (PNH) is a rare disorder of hematopoietic stem cells, characterized by hemolytic anemia, bone marrow disorders, and thrombophilia [1]. A somatic mutation on the PIG-A gene is the trigger for the development of PNH. The PIG-A gene participates in the early step of glycosylphosphatidylinositol (GPI)-anchor biosynthesis [2].

The lack of one of the GPI anchored proteins (GPI-AP), like complement regulatory proteins CD55 and CD59, leads to the hemolysis of red blood cells (RBCs). The destruction of these cells is the main reason for the symptom panoply related to this disorder, evidenced especially when the clonal expansion of the PIG-A mutant stem cell is high and which leads to relevant clinical manifestations [3]. The hemolytic state of PNH relates to expected fatigue but also to dyspnea, dysphagia, and abdominal pain [4]. The cause and pathological background of the clonal expansion of the mutated cells have not yet been elucidated [5].

Life-threatening thrombosis is the most feared outcome of PNH. Besides the absence of complement-regulating surface proteins, many mechanisms can be associated with thrombosis susceptibility, like platelet activation, free hemoglobin (Hb) levels, nitric oxide depletion, endothelial dysfunction, and the loss of the GPI-anchored urokinase receptor, which interferes with fibrinolysis [6,7,8].

Thrombotic events may appear at unusual locations and with no unanimous scientific explanation. Thrombosis in visceral veins is frequently reported like hepatic (Budd–Chiari syndrome), mesenteric, portal, splenic, and inferior vena cava. Pulmonary embolism, cerebral, renal, and dermal vein thrombosis can also occur [9,10].

The great advancement in PNH prognosis and treatment management was the discovery of the monoclonal antibody (mAB) eculizumab, which inhibits the complement cascade, blocking C5 and preventing terminal complement assembly. Complement inhibition had a major impact in reducing intravascular hemolysis and in the prevention of thrombosis as well as in controlling this disease [11].

During the COVID-19 pandemic, physicians were concerned about a possible worse outcome for infected PNH patients. Patients with a high risk of thromboembolism, and the association of abnormal coagulation parameters are usually related to poor prognosis in patients with coronavirus pneumonia [12]. Complement activation may contribute to severe acute respiratory syndrome coronavirus (SARS-CoV) pathogenesis [13]. Studies are underway to evaluate eculizumab as an agent to prevent severe disease during SARS-CoV-2 infection, which would lead to a good prospective outcome for previously eculizumab-treated patients, while also keeping in mind the great prognostic variability in individuals infected with COVID-19 [14].

## 2. Detailed Case Description

The patient is an active 41-year-old Caucasian male with a 15-year history of classical PNH with a year-older brother as a suitable donor for allogenic bone marrow transplantation.

The patient has a healthy lifestyle and is in a good familiar and psychosocial environment. Clinically, the patient had a previous history of varicocele surgery and appendix surgery.

On first admission, he reported easy bruising, extreme asthenia, and myalgia. He exhibited unexplained cytopenia and hemolytic anemia confirmed by high levels of lactate dehydrogenase (LDH). The direct antiglobulin test was negative. HIV and hepatitis tests were negative. Serum IgG and IgM of cytomegalovirus (CMV) were elevated. Bone marrow aspirate did not evidence any signs of malign transformation. PNH screening by flow cytometry revealed a deficiency of GPI-anchored proteins on multiple cell lineages, establishing the diagnosis of PNH.

For 11 years, his disease manifested with chronic hemolysis, hemoglobinuria, fatigue, and occasional paroxysms of abdominal pain.

His treatment consisted of irradiated RBC transfusion. Initially, he had few exacerbations of the disease but gradually the frequency of the flares increased.

He was treated with folic acid supplementation and intermittent pulses of prednisolone, especially in the first year after diagnosis.

**Figure 1:**
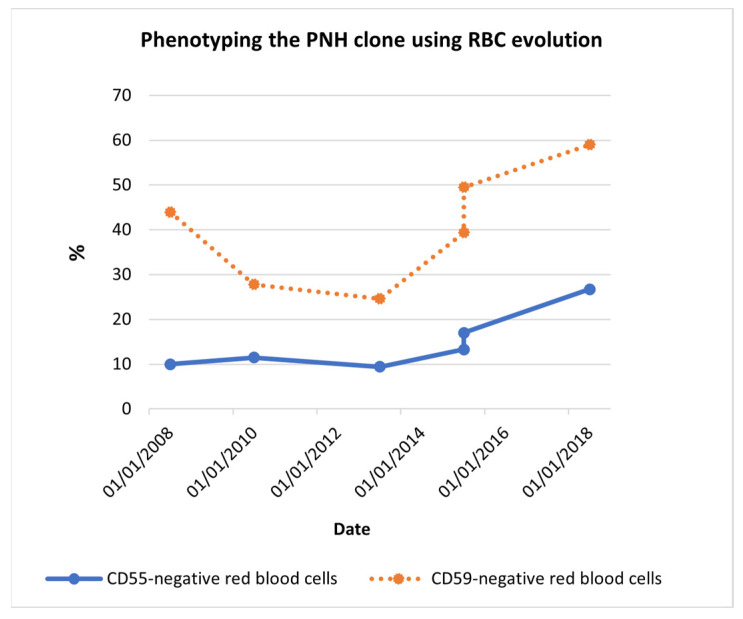
Phenotyping the PNH clone using RBC evolution. Red blood cell phenotyping data showed, at the time of diagnosis (2008), 10.0% CD55^−^ RBCs and 44.0% CD59^−^ RBCs. Despite the decrease seen between 2008 and 2013, 10 years later, CD55^−^ RBCs became 26.7% and CD59^−^ RBCs became 59.0%. The PNH clone increasingly expanded among the years. Abbreviations: CD55^−^ RBCs, CD55-negative red blood cells; CD59^−^ RBCs, CD59-negative red blood cells.

Ten years after the diagnostic testing, the transfusion requirement increased from one or two times per month to one or two times a week. Over 10 years, the PNH clone expanded, as phenotyping data proved (Table 1 and Figure 1).

**Figure 2:**
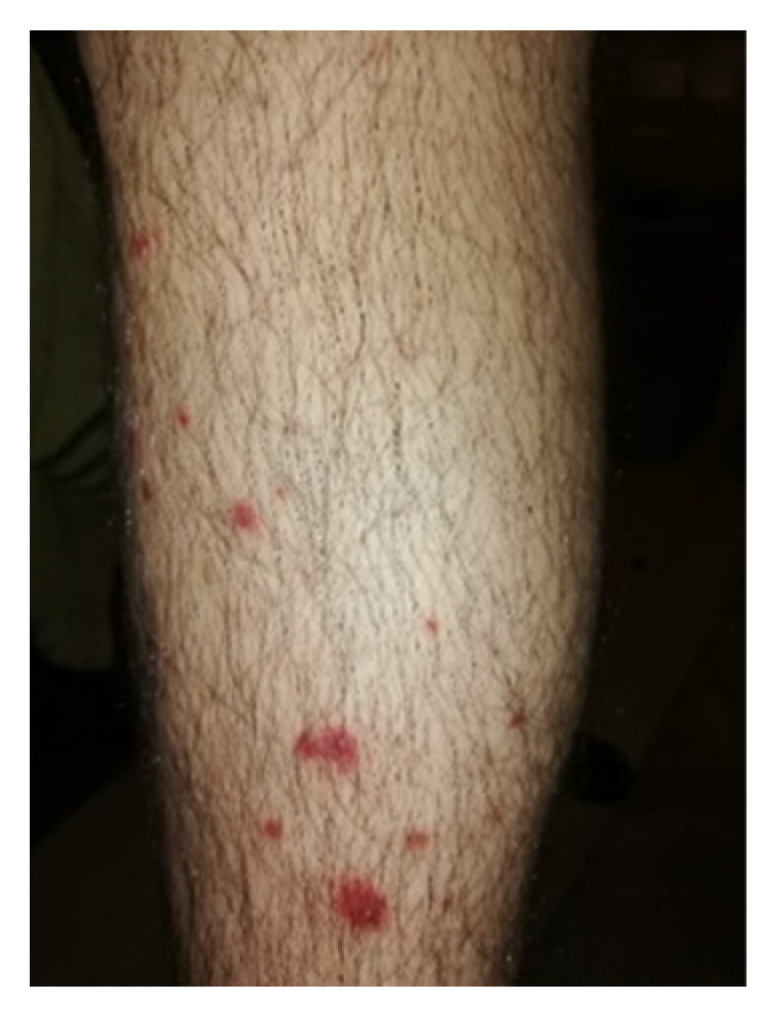
Leg dermal vein lesion. The lesions appeared punctually, probably caused by dermal thrombosis and/or vasculitis. The lesions eventually spread and formed a wound with soft tissue infection, which were treated with antibiotics.

Ten years after the diagnostic testing, he started to exhibit dermal lesions on his inferior limbs with the appearance of dermal vein thrombosis (Figure 2).

Table 2 shows clinical data from the last two and a half years before the introduction of the eculizumab treatment:

Hemoglobin levels were always under 9.0 g/dL (reference range 13.0–18.0) even with the best supportive therapy and with the high rate of RCB transfusion.

Leucocytes and platelets levels are under the minimum level (reference range 4.0 × 10^9^/L and 140 × 10^9^/L, respectively), supporting the pancytopenia state.

The LDH reference values are 135–225 U/L and the LDH levels ranged from 7 to 10 times U.L.N. (upper limit of normal), supporting a hemolytic state.

The C-reactive protein (CRP) reference value is under 3.0 mg/L and the patient’s levels were always higher than this value.

The worsening of symptoms, higher hemolysis (evidenced by extremely high values of LDH) and anemia, as well as the increase in transfusion frequency, led to the request, by the physician, for eculizumab treatment.

While waiting, the patient started the vaccine protocol. It is an important and necessary step to prevent opportunistic infections by Neisseria and other pathogens during anti-complement therapy. The first phase protocol included Prevenar^®^ (pneumococcal polysaccharide conjugate vaccine) and Hiberix^®^ (Haemophilus influenzae b conjugate vaccine) administrated on the same day followed 18 days later by Nimenrix^®^ (meningococcal groups A, C, W-135, and Y conjugate vaccine) and Bexsero^®^ (meningococcal group B vaccine) (Table 3).

After the first phase of the vaccination protocol, the patient started to experience excruciating abdominal pain and reported the appearance of dark urine. He also developed a fever and could not eat properly.

The abdominal pain increased and became unbearable. Then, on 3 July 2019, 22 days after the first phase of vaccination, the patient was diagnosed with splenic vein thrombosis.

**Figure 3:**
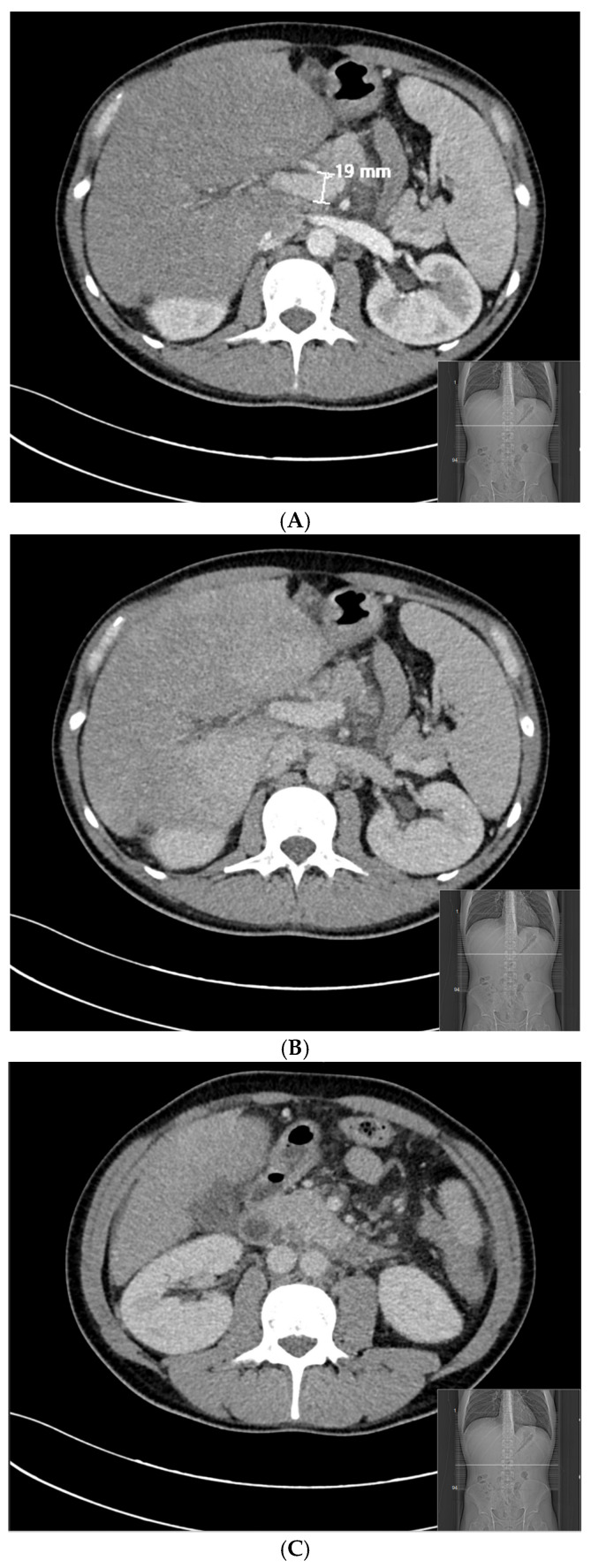
Abdominal CT angiography 23 days after the patient’s splenic vein thrombosis. (**A**) (arterial phase) and (**B**) (venous phase): the splenoportal axis is contrasted and dilated up to the confluence with the superior mesenteric vein. (**C**) (venous phase): after the superior mesenteric vein, it was not possible to identify the course of the splenic vein.

Twenty-three days later, after the initiation of the anticoagulant treatment, abdominal CT angiography was repeated and showed that the splenoportal axis was contrasted and dilated up to the confluence with the superior mesenteric vein, from which it was not possible to identify the course of the splenic vein, due to the occurrence of thrombosis (Figure 3). Over a period of two months of acute symptoms, the patient lost 11 kg of weight.

The patient was placed on anticoagulation with low-molecular-weight heparin and then converted to warfarin therapy. His clinical condition progressively meliorated despite of difficulty maintaining a therapeutic INR in the first months.

To avoid an important outcome, the patient asked to put off the vaccine protocol and it resumed only six months after the first phase.

Eculizumab was initiated three months after the splenic stroke, which resulted in an immediate reduction in intravascular hemolysis, evidenced by a decrease in LDH. LDH decreased from 2324 U/L during the flare (2019) to 360 U/L (2022) within 30 months after initiation of eculizumab (900 mg IV, every 14 days).

**Figure 4:**
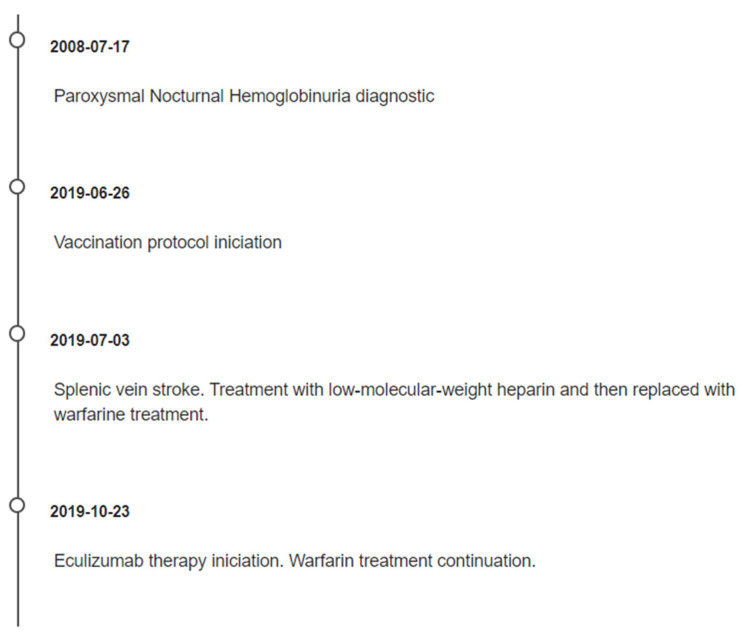
Case study timeline.

The case study timeline is presented in Figure 4.

The patient experienced a decreased need for RBC transfusions. He required only two RBC transfusion since starting the mAB. RBC transfusion was needed after the second dose of Bexsero^®^ and after Pneumovax^®^ (pneumococcal polysaccharide vaccine). After the second dose of Nimenrix^®^, the patient did not experience breakthrough hemolysis.

Treatment with eculizumab continued and there was no evidence of overt hemolysis or complement-mediated breakthroughs. We could say that the patient became transfusion-independent, with LDH levels near 1.5 U.L.N. (upper limit of normal). He has not experienced any other thrombotic event.

Anemia persisted, with hemoglobin levels near 10 g/dL and signs of thrombocytopenia, mainly after the eculizumab and warfarin treatments. Eculizumab has been shown to be effective in preventing thromboembolism. Physicians maintained treatment with warfarin and with eculizumab.

Table 4 displays analytical results: (a) after the first phase of vaccination; (b) after the anticoagulant therapy initiation; (c) after the eculizumab initiation; (d) after the second dose of Bexsero^®^; (e) after the recovery of the vaccination outcome.

The patient attended every scheduled treatment at the hospital and participated in all the follow-up laboratory tests. He revealed a significant improvement in the symptoms of the disease.

During the global pandemic of COVID-19, the patient was infected but did not develop major symptoms. Besides the loss of taste and smell, he experienced slight asthenia, which is usually related to PNH.

Due to this infection, the eculizumab schedule was delayed 7 days, so the interval of doses was 21 days, instead of 14, but it did not cause any hemolytic crisis to develop, as could be expected.

The patient was infected in November of 2020, before COVID-19 vaccination. The vaccine administrated was Comirnaty^®^ (mRNA SARS-CoV-2 vaccine): the first dose was in 2 July 2021 and the second dose was in 1 February 2022. The patient has not shown any vaccination side effects.

## 3. Discussion

Thrombotic events can be lethal and a patient with PNH has a major propensity to thromboembolism. This case emphasizes the importance of individual therapeutic and preventive management, according to the variable aspects of this disease. Severe difficulties to the access of some of the therapies should not be an obstacle to better care and a better prognosis for the disease.

A systematic review associated hemolytic anemia, thrombocytopenia, pancytopenia, and other hematological disorders with CMV infection [15]. The PNH clone may have expanded during the CMV primary infection. The pathogenesis is still unclear, although immunologic mechanisms may be involved [16,17,18].

Increasing hemolysis and the number of RBC transfusion can be associated with higher clonal evolution, but it may occur in patients with smaller clones [19]. It is important to identify the burden and the activity of the disease to initiate therapy (anticoagulant and/or anti-complement) before major thrombotic events occur [20]. However, primary prophylaxis can reduce the risk, but it does not completely prevent thrombosis [21]. A prior thrombotic event may require anticoagulant therapy with warfarin and others, but this type of prevention may not be efficient in the long term [22].

As far as we know, the appearance of thrombosis in PNH can be a combination of complement activation with hemolysis [23]. Vaccine administration can lead to extreme complement activation, which can be a large problem for PNH patients. There are some cases reported with hemolytic states appearing after infection with COVID-19 and the administration of the influenza vaccine and their relationship with PNH [24,25,26]. Vaccines can trigger immune responses in the body, which might lead to systemic side effects. In the case of concomitant administration vaccines, the production of high levels of inflammatory mediators is associated with stronger immune responses. A common adverse event is febrile illness, and the frequency of high fever rises when multiple vaccines are administered simultaneously [27,28].

This patient exhibited a peak of grave symptoms after the first phase of vaccination (Table 4a), which was probably caused by extreme complement activation. However, the complement activation was not directly measured.

The hemoglobin level dropped and the patient had a big increase in the need for transfusion support and was treated with prednisolone. The rise in the C-reactive protein, as an early indicator of infection or inflammation, also supports the acute state of the disease [29]. Hemolytic anemia was very active. The massive destruction of erythrocytes may be associated with splenic vein stroke, causing severe abdominal pain [30].

Vaccine-induced thrombosis and its potential mechanisms were under major scientific research after COVID-19 vaccination. However, the mechanisms that induce thrombosis with other kinds of vaccines are rarely mentioned in the literature [31].

In this case, we believe that dysregulation of the complement system was the major trigger of the thrombotic event. The complement cascade is directly connected to the activation of proteases of coagulation and fibrinolytic systems [32]. Complement activation can lead to platelet stimulation and to endothelial cell activation while the membrane attack complex is linked to tissue damage [33,34].

As far as we know, it is the first time that a relationship has been reported between eculizumab vaccination protocol and a thrombotic event.

Studies prove that eculizumab prevents thrombotic events and the discontinuation of anticoagulation therapy after eculizumab administration is an option that can be studied [35].

The maintenance of anemia may be evidence of extravascular hemolysis. Eculizumab compensates for the deficiency of CD59 and prevents intravascular hemolysis; however, RBC loss of CD55, which inhibits the C3 and C5 convertases upstream to C5, leads to the accumulation of C3 fragments. The PNH erythrocytes of some patients become coated with these C3 fragments, which leads to extravascular hemolysis [36]. Trials with advanced new therapeutic drugs are trying to quell the onset of extravascular hemolysis [37].

COVID-19 is caused by the SARS-CoV-2 infection. In severe cases, inflammation and coagulation states are correlated with hyper-activation of complement cascades and cytokine exacerbation. If the patient reaches the inflammatory phase, eculizumab may be considered as a therapeutic option, as well as heparin [38].

Some studies support that administration of the anti-C5 monoclonal antibody eculizumab and other anti-C5 drugs led to a rapid and marked decline in biomarkers, for systemic clotting and inflammation in critical COVID-19 patients, decreasing the cytokine cascade and ameliorating respiratory symptoms, as well as decreasing the events related to thrombosis and the eventual associated organ damage [39,40,41,42].

As a potent inhibitor of complement activation, eculizumab can reduce immunity and improve infection susceptibility, making this drug part of the list of possible suitable prophylaxis drugs to prevent SARS-CoV-2 infection.

## 4. Conclusions

There is still a lot to understand concerning the clonal expansion of the PIG-A clone. The patient’s recent CMV infection could be a trigger for the clonal evolution, as it was previously related to other hemolytic anemias.

In this clinical case, when the patient was naïve to complement inhibitor treatment, the massive hemolysis due to exacerbated complement activation was probably caused by vaccine administration, causing a vaccine-induced thrombosis.

The use of eculizumab transformed this patient’s live and reduced the propensity of thrombotic events. It represented an improvement in clinical parameters and the patient became transfusion independent.

This patient case report is evidence of anti-complement therapeutics benefits, not only because of improvements in quality of life and symptoms of PNH but also for the possible protective effect it had in preventing the worst outcome in an infection of COVID-19.

### Patient Perspective

“Having PNH made me feel special, different, angry, and persevering. I could not give up or stop. The first symptoms were scary. I didn’t get out of bed, I just slept! I had a lot of muscle and joint pain… I felt powerless. It was difficult to accept the disease and I was stubborn to adapt to its limitations. I have done many RBC transfusions that made me feel strong, but the risks were always on my mind. I always thought maybe one day it wouldn’t go so well. The new treatment was important! I believed that I was going to have an eculizumab prescription, but it was a lengthy process that ended in a thrombosis after the vaccines. Very tough days! I thought I was going to die, but I believed in a solution, and hopefully I had eculizumab in my veins, like an elixir of eternal life. Every 15 days I went for my “moment” and felt rejuvenated. I started running again, I stopped having so much muscle pain... I was able to get back to having a life. Now I feel even more special… after all I got this precious treatment. And life goes on”.

## Figures and Tables

**Table 1:** Phenotyping the PNH clone using RBC evolution.

Date	% CD55^−^ RBCs	% CD59^−^ RBCs
17 July 2008	10.0	44.0
30 April 2010	11.5	27.8
9 May 2013	9.4	24.6
22 April 2015	13.3	39.4
29 September 2015	17.0	49.5
5 September 2018	26.7	59.0

Abbreviations: CD55^−^ RBCs, CD55—negative red blood cells; CD59^−^ RBCs, CD59—negative red blood cells.

**Table 2:** Clinical data with supportive therapy.

Date	Hb (g/dL)	Leucocytes (10^9^/L)	Platelets (10^9^/L)	LDH (U/L)	CRP (mg/L)
31 May 2017	8.1	3.14	121	1870	8.1
26 December 2018	7.8	3.38	131	1674	16.0
20 February 2019	8.8	3.98	107	1826	48.9
30 March 2019	8.5	2.84	119	2301	13.3
8 May 2019	6.8	2.65	113	2091	8.3

Abbreviations: Hb, hemoglobin; LDH, lactate dehydrogenase; CRP, C-reactive protein.

**Table 3:** Vaccination protocol.

Date	Vaccine Administration
**1st phase**	
24 May 2019	Prevenar^®^ and Hiberix^®^
11 June 2019	Nimenrix^®^ (1st dose) and Bexsero^®^ (1st dose)
**2nd phase**	
10 December 2019	Bexsero^®^ (2nd dose)
23 January 2020	Pneumovax^®^
2 March 2020	Nimenrix^®^ (2nd dose)

**Table 4:** Clinical data after initiation of vaccination, anticoagulant therapy, and after eculizumab.

Date	Hb (g/dL)	Leucocytes (10^9^/L)	Platelets (10^9^/L)	LDH (U/L)	CRP (mg/L)
**(a) After 1st phase vaccination**					
26 June 2019	5.8	3.32	113	1343	72.9
3 July 2019	7.3	3.31	104	1847	63.6
**(b) After anticoagulant therapy**					
9 October 2019	8.7	4.08	82	2324	71.8
**(c) After eculizumab initiation**					
29 October 2019	8.0	3.27	99	1123	11.6
13 November 2019	9.3	3.23	107	532	5.8
20 November 2019	9.2	3.18	99	486	11.2
3 December 2019	8.6	5.10	89	401	22.9
**(d) After Bexsero^®^ (2nd dose)**					
17 December 2019	6.8	5.28	98	568	76.1
**(e) After the recovery of the vaccination outcome**					
26 December 2019	10.2	3.19	124	428	7.6
15 January 2020	10.6	4.29	109	441	4.8
20 May 2020	10.2	3.18	89	405	7.4
18 June 2020	10.3	2.58	90	328	6.5
11 November 2021	10.1	2.91	104	394	6.5
22 April 2022	9.9	3.01	108	360	6.0

Abbreviations: Hb, hemoglobin; LDH, lactate dehydrogenase; CRP, C-reactive protein.

## Data Availability

Not applicable.
